# Genomic analysis of carbon dioxide sequestering bacterium for exopolysaccharides production

**DOI:** 10.1038/s41598-019-41052-0

**Published:** 2019-03-12

**Authors:** Manish Kumar, Madan Kumar, Ashok Pandey, Indu Shekhar Thakur

**Affiliations:** 1 School of Environmental Sciences, Jawaharlal Nehru University, New Delhi, 110067 India; 2 CSIR-Indian Institute of Toxicology Research, 31 MG Marg, Lucknow, 226 001 India

## Abstract

In the present study, genomic analysis of a previously reported carbon dioxide (CO_2_) sequestering bacterium *Serratia* sp. ISTD04 was performed along with exopolysaccharide (EPS) production. Genomic analysis identified key and accessory enzymes responsible for CO_2_ sequestration. EPS synthesis genes were discovered in the genome and identified 8 putative clusters responsible for lipopolysaccharide, stewartan, emulsan, polysaccharide B, capsular polysaccharide and fatty acid-saccharide production. The production of EPS was found to be 0.88 ± 0.08, 1.25 ± 0.13 and 1.44 ± 0.10 g L^−1^ on glucose, bicarbonate (NaHCO_3_) and NaHCO_3_ plus glucose respectively at pH 7.8. After optimizing process parameters, the EPS production increased more than 3 folds. The morphology of strain and elemental composition of EPS was characterized by SEM-EDX. The functional groups, monomer composition, linkage analysis and structure of purified EPS was characterized by FTIR, GC-MS and ^1^H and ^13^C NMR. Glucose, galactose, mannose and glucosamine are the monomers detected in the EPS. EPS was further applied for bioflocculation (kaolin test) and dye removal. The EPS showed 68% ± 0.9 flocculating activity and decolorized cationic dye acridine orange (80%) and crystal violet (95%). The results highlight CO_2_ sequestration and EPS production potential of *Serratia* sp. ISTD04 that can be harnessed in future.

## Introduction

With the increase in atmospheric carbon dioxide (CO_2_) concentration, there is disturbance in global climate equilibrium as a result the global temperature is rising at a continuous pace. This increase in CO_2_ emission is mainly due to unchecked anthropogenic activities. So, there is an urgent need to reduce the level of CO_2_ emission in the atmosphere from various sources. The emission can be reduced by various physical, chemical and biological processes^1,2^. Biological fixation of CO_2_ by plants and microorganisms is the most common and effective process for sustainable CO_2_ sequestration^2^. The microorganism accountable for CO_2_ sequestration belongs to archaea (*Euryarchaeota* and *Crenarchaeota*) and bacteria (*Aquificae, Actinobacteria, Chloroflexi Proteobacteria, Chlorobi, Firmicutes* and *Thermodesulfobacteria*). Microbes can fix CO_2_ through six known pathways but the most predominant is Calvin–Benson–Bassham (CBB) pathway^2,3^. The microorganism sequesters CO_2_ through a carbon concentrating mechanism with the help of well-known enzymes Ribulose-1,5-bisphosphate carboxylase/oxygenase (RuBisCO) and carbonic anhydrase^3^. These microbes can convert CO_2_ into biomass and bioproducts such as lipids, polyhydroxyalkanoates (PHAs), and extracellular polymeric substances (EPSs)^2,4,5^. *Serratia* sp. ISTD04 has been reported for CO_2_ sequestration and production of value- added products^1,5^.

Genus *Serratia* belongs to *Enterobacteriaceae* family; they are gram-negative, rod-shaped and facultative anaerobes that dwells in a diverse environment such as water, soil, plants, rhizospheric soil and other organisms^6^. *Serratia* has been reported from diverse site and perform various functions such as *Serratia fonticola* RB-25 isolated from a waste landfill for quorum sensing, *Serratia plymuthica and other* strains AS12, AS9, S13 and 4Rx13 are associated with plants or plant-growth-promoting activities, *Serratia proteamaculans* 568 with a detailed genome analysis on chitinase production, *Serratia marcescens* WW4 isolated from a paper machine, *S. marcescens* FGI 94 associated with leaf-cutter ant fungus garden and *S. marcescens* Db11 pathogen of drosophila^4,7,8^. This genus is also known for production of value-added products such as enzymes, biosurfactants, pigments, fatty acids, flavors and polyhydroxyalkanoates^2,3,9^. The genes and pathways responsible for CO_2_ sequestration and bioproduct synthesis can be further elucidated by the genomic, transcriptomic and proteomic approach. Identification of genes and enzymes controlling particular process will provide an opportunity to further improve the process through genetic engineering approach^2,3,5^. There are relatively few strains of *Serratia* genus has been sequenced compared to *Escherichia* and *Salmonella*^7^. The genomic analysis will provide information and highlight the diverse capability of this genus.

Biopolymers are composed of a natural resource that can be produced sustainably from renewable sources and are completely biodegradable. These polymers form a protective structure around microbes, acts as reserve material and also provide adaptability to uncertain environmental conditions^10^. The microorganisms can convert diverse carbon sources and synthesize both extracellular as well as intracellular biopolymers. The majority of polymers produced are extracellular and very few intracellular. The bacteria produce several types of biopolymers that can be categorized into four major class polysaccharides, polyamides, inorganic polyanhydrides and polyesters^2,10^. The bacterial EPS can be classified as exopolysaccharide, capsular polysaccharide and intracellular polysaccharide^11^. Depending upon the linkage and monomeric units; the polymer can be grouped into homopolymer (α-d-glucans, β-d-glucans, fructans) or heteropolymer (*N*-acetylglucosamine (GlcNAc), *N*-acetylgalactosamine (GalNAc), or glucuronic acid (GlcA) and d-glucose, d-galactose, l-rhamnose,) with presence of other substances such as lipid, protein, nucleic acids, acetyl group and phosphate^12,13^. The presence of different sugar residues provides different structures and conformations to EPS^14^. Bacterial strains such as *S. marcescens*, *Aeromonas salominicida*, and *Pseudomonas* sp. strain NCIB 2021 have been reported for heteropolysaccharide production^13^. The strains, *S. marcescens, Aeromonas salominicida* and *Pseudomonas sp*. strain NCIB 2021 are known to produce two different polysaccharides^13^. The synthesis of EPS monomers takes place inside or on the cell surface with the coordinated regulation of genes and action of enzymes. The genes for synthesis, assembly and export of EPS are generally present in the form of clusters and the genes in clusters are tightly regulated^13,15^. There are several reports on identification and isolation of different types of EPS from diverse sources such as bacteria, fungi, microalgae and plants^14^.

Based on their chemical composition and nature, there are several applications of EPS such as bioflocculation, provide defense against various toxin, avoid host immune response, limits penetration of various substance, antimicrobial and antibiofilm activity, dye decolorization and reclamation of waste lands^12,14,16^.

The bacterial strain ISTD04 was previously isolated and characterized in detail for its CO_2_ sequestration potential along with lipid and PHA production^3,5,9^. The genome of *Serratia* sp. ISTD04 was sequenced earlier and the sequence is openly available with NCBI accession number MBDW00000000.1^17^. The detail proteomics (2D-GEL) and nano-LC-MS/MS of *Serratia* sp. ISTD04 has been done for identification of enzymes differentially regulated during CO_2_ sequestration and biodiesel production^3,5^. Comprehensive analysis of genome from *Serratia* sp. ISTD04 will complete the proteomic findings as well as identify novel genes and clusters responsible for bioproducts synthesis. In this study, we have comprehensively analyzed the *Serratia* sp. ISTD04 genome. The bioinformatics analysis was done to discover putative genes and pathways responsible for CO_2_ sequestration and synthesis of bioproducts such as EPS, lipids, PHA. Thereafter, important gene clusters responsible for EPS production were also highlighted. The production and characterization of EPS by *Serratia* sp. ISTD04 in presence of NaHCO_3_ as carbon source and glucose as inducer was carried out. In addition, we have also demonstrated the environmental application of EPS.

## Result and Discussion

### Genomic analysis of *Serratia* sp ISTD04 for CO_2_ sequestration and synthesis of bioproducts

**Figure 1:**
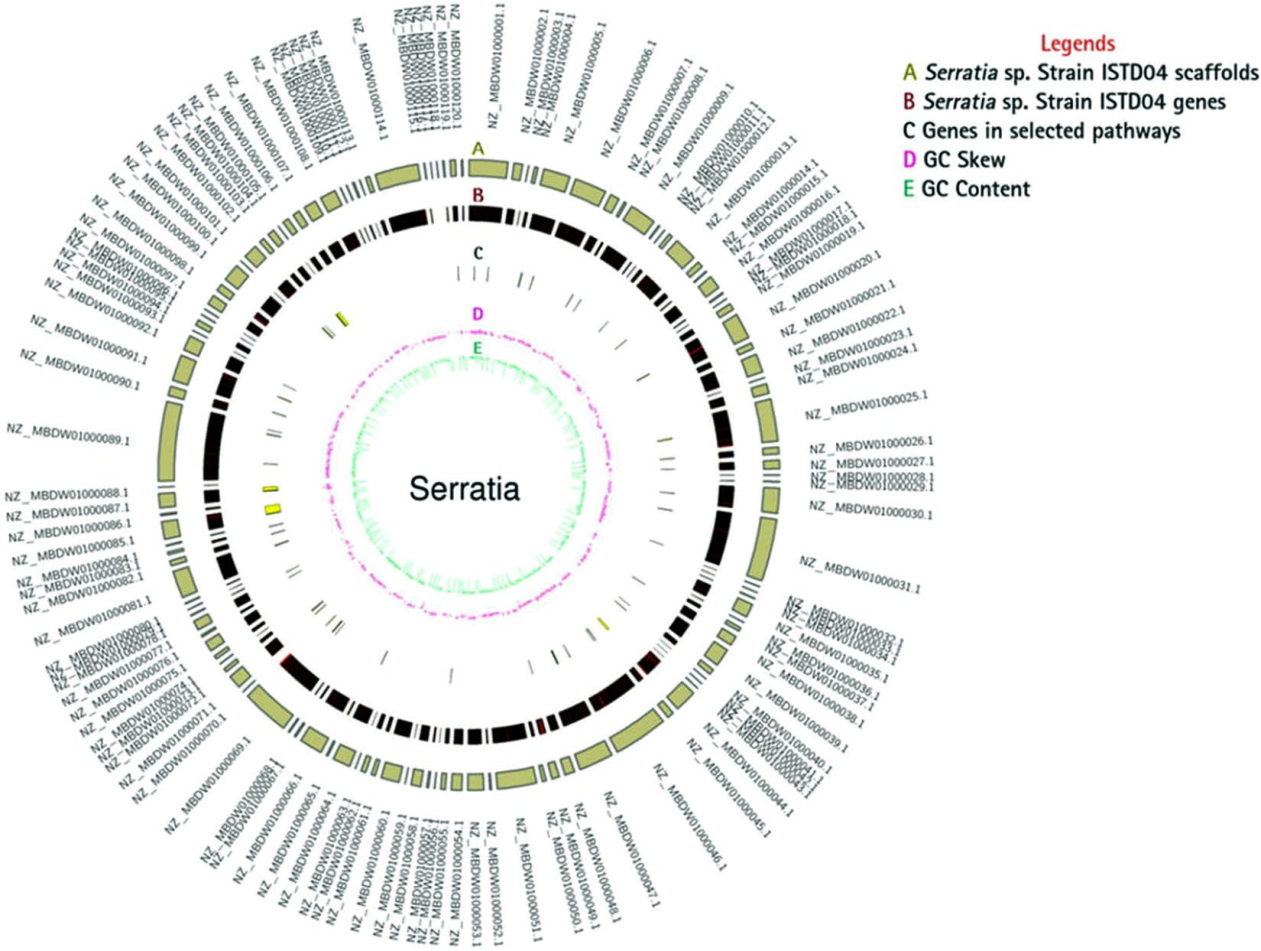
Circos representation of genes compared with the genome for *Serratia sp. ISTD04*. Circles from outside to inside represent; (**A**) scaffold arrangement, (**B**) gene position on the scaffolds; (**C**) genes in selected pathways (**D**) GC skew; and (**E**) GC content. Detail of genes in selected pathway is represented in Table S1.

The *Serratia* sp. ISTD04 was previously characterized for CO_2_ sequestration along with the production of lipids and polyhydroxyalakanoates^3,5,9^. The genome size of *Serratia* sp. ISTD04 is 5.07 Mb with 81X coverage having GC content of 59.98%, 4,563 predicted protein-coding genes (Prokaryotic Genome Annotation Pipeline (PGAP) and Pfam annotation) and the other general genome features have also been reported earlier^17^. A Circos plot showing space efficient and clear representation of genes on the genome and the important genes and their position related to CO_2_ sequestration and EPS production have been shown on the circular plot (Fig. 1 and Table S1).

The ‘nr’ blast of important proteins responsible for CO_2_ sequestration and EPS production is represented in Table S2. Genomic analysis of *Serratia* sp. ISTD04 shows that all the important genes and proteins of CBB pathway are present in this strain but RuBisCO is missing in the sequence^17^. Genomic investigation of *Serratia* sp. ISTD04, substantiate its diverse metabolism and clearly depicted that this strain follows CBB cycle for the sequestration of CO_2_^17^. Among the 13 known enzymes of CBB pathway, RuBisCO and phosphoribulokinase (PRK) are the two key enzymes, RuBisCO is missing but PRK and other 11 enzymes are annotated in the genome. Carbonic anhydrase, a facilitator in CO2 fixation was annotated and present in multiple copies in the genome. Along with RuBisCO and carbonic anhydrase, the other enzymes such as FBPase and two aldolases were also detected that plays important role in CBB cycle. *Serratia* sp. ISTD04 is a chemoautotrophic bacterium and in addition to CO_2_ this strain can metabolize monosaccharide (galactose, mannose, and fructose), disaccharides (sucrose) polysaccharides (starch) and many organic compounds such as, glucuronate, ascorbate, aldarate, amino sugar, nucleotide sugar, propionate, butanoate, glyoxylate, dicarboxylate and pyruvate as carbon source. Since this is a draft genome so there is a possibility that the RuBisCO gene may not be sequenced or annotated in the genome. The proteomic analysis of this strain discovered both large and small subunit of RuBisCO and it was further confirmed by western blotting. The PRK and other enzymes actively involved in CBB cycle were also detected in the proteomics^3,5^. The carboxylation property of RuBisCO is maintained in a specific micro-compartment i.e., carboxysome and carbonic anhydrase perform interconversion (HCO^3−^ to CO_2_ and vice versa) and make CO_2_ available for RuBisCO^2,3^. PRK, an octameric protein with size 32–36 kDa execute the final step in RuBP generation was also reported from other photosynthetic bacteria such as *Ralstonia eutropha, R. acidophila*, and *Rhodobacter sphaeroides*^18–20^. In *Xanthobacter flavu*s the aldolases were reported both in heterotrophic and autotrophic condition and the aldolase activity increased 14-fold during autotrophic condition^21^. The presence of both the aldolases in *Serratia* sp. ISTD04 confirmed that this strain can perform both autotrophic as well as the heterotrophic mode of growth, depends on the availability of diverse groups of substrate. The key and accessory enzymes present in *Serratia* sp. ISTD04 validates its CO_2_ fixation potential through CBB pathway.

### Identification of EPS Cluster in *Serratia* sp. ISTD04

**Figure 2:**
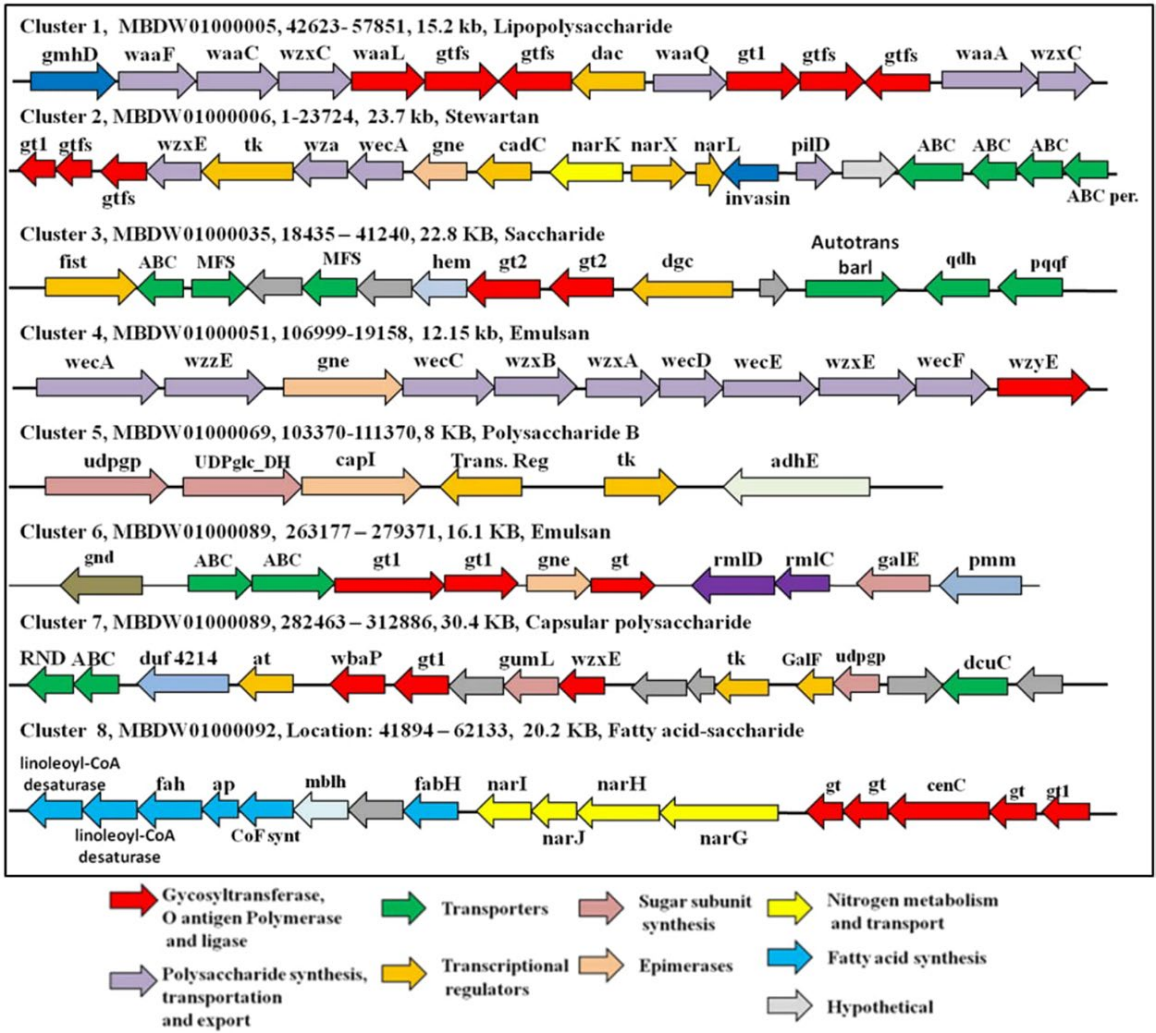
Represents putative gene clusters with contig number, position and size identified in *Serratia* sp. ISTD04 genome responsible for polysaccharide production.

EPS synthesis, regulation, polymerization and export-related genes are dispersed throughout the genome of *Serratia* sp. ISTD04, but there are eight major clusters discovered that are responsible for capsular polysaccharide, Polysaccharide B, stewartan, emulsion, saccharide, lipopolysaccharide, and fatty acid-saccharide production. The EPS gene clusters with contig and position details from *Serratia* sp. ISTD04 has been represented in Fig. 2. The cluster 2 (stewartan), 3 (saccharide), 5 (polysaccharide B) and 7 (capsular polysaccharide) showed less than 15% similarity with *Pseudomonas cichorii* JBC1, *P. fuscovaginae* strain IRRI 6609, *Xenorhabdus nematophila* str. *Websteri* and *Burkholderia* sp. RPE64 respectively at genes level. Cluster 1(lipopolysaccharide), 4 (emulsan), 6 (emulsan), and 8 (fatty acid-saccharide) showed 30%, 28%, 19% and 36% sequence similarity with *Yersinia ruckeri*, *Y. frederiksenii*, *P. putida* LS46 and *P. syringae* strain UB0390 respectiveely. The presence of polysaccharide synthesis, polymerization and export-related genes such as wzy, wzx, wzc, wza, wzz, wca, glycosyltransferases and transporters were observed in the cluster. The presence of an additional enterobacterial common antigen polymerase (wzy) was discovered in the genome away from the cluster on scaffold 8. In addition, genes responsible for nucleotide sugar precursor synthesis were discovered in the cluster as well as found dispersed throughout the genome. The cluster 2 and 8 include genes for the nitrate/nitrite dependent regulation. The other regulators such as tyrosine kinase, GalU regulator GalF, diguanylate cyclase, sigma-54-dependent Fis family transcriptional regulator, two-component system regulator and other transcriptional regulators were discovered in the identified clusters. Furthermore, all the above-identified putative clusters showed similarity with the members of *Enterobacteriaceae* family indicating that clusters are conserved and specific to this family. The Cluster 8 (fatty acid-saccharide cluster) was unique to this strain and this cluster contains nitrogen metabolism gene flanked by fatty acid and polysaccharide synthesis genes. The cluster analysis supported the GC-MS of monosaccharide composition as the genes for the production of glucose, mannose, galactose and GalNAc were identified.

The mechanism of EPS production in bacteria is known to occur via four pathways such as Wzx/Wzy-dependent pathway, ABS transporter-dependent pathway, synthase-dependent pathway and extracellular single sucrase protein^15^. In Gram-negative bacteria Wzx/Wzy-dependent and ABC-transporter dependent pathways are usually observed for EPS production. The presence of transporters (ABC, RND, MFS and autotransporter), glycosyltransferase (gts) and O-antigen polymerase (wzy) in clusters point towards both Wzx/Wzy-dependent and ABC-transporter dependent pathways for EPS production. In Wzx/Wzy-dependent pathway the assembly of repeating units on lipid carrier occurs on the cytoplasmic face. The carrier with repeating unit gets flipped towards periplasmic face by Wzx (flippase) and the Wzy (O-polymerase) add the repeating units to the polymer. The polymers produced by this pathway contain diverse sugar components and results in heteropolysaccharide production^15^. The presence of flippase or polymerase i.e., Wzx, Wec and Wzy in cluster 1, 2 4 and 7 indicates Wzx/Wzy-dependent pathways for synthesis and export of EPS. The absence of polymerase gene in strains 215W4-47a and NRBB56 linked EPS cluster was observed but still they are able to produce EPS^22^. ABC-transporter dependent pathways are mainly involved in the synthesis of capsular polysaccharide^15^. In transporter-mediated pathways the synthesis of polysaccharide takes place towards the cytosolic face and the GTs elongate the polysaccharide chain at the non-reducing end followed by its transportation across cytoplasmic membrane by ABC transporters^23^.

Cluster 3 and 6 contain GTs, transporters and lack Wzx, Wzc and Wzy indicating ABC-transporter dependent pathways. Since multiple GTs observed in the identified cluster so the polymer produced is heteropolymer^15^. Wzc proteins are member of polysaccharide copolymerase 2a family involved in the synthesis of high molecular weight EPS and CPS. Wzc protein deletion affects assembly of repeating units to the polymer. The detection of UDP-phosphate galactose phosphotransferase (wbaP) in the cluster 7 point towards the presence of galactose as initiating sugar as observed in the case of *Salmonella* O antigens. The WbaP has been shown to have both galactosyltransferase and flippase function. Wzz (polysaccharide chain length modulation protein) regulates translocation of the subunits in an ATP hydrolysis-independent manner and the chain length is regulated via DNA adenine methyltransferase (Dam) of surface polysaccharide. Wzz are member of polysaccharide copolymerase 1 family and act as a ruler for polysaccharide chain length synthesis. Lack of Wzz resulted in unregulated chain length and its characteristics. O antigen ligase (WaaL) transfers the O-antigen monomer and polymer to lipopolysaccharide precursor (lipid A-core) as a result completing lipopolysaccharide synthesis along with the release of lipid carrier. This WaaL protein has also been shown to regulate the O-unit synthesis based on availability in the periplasmic side by feedback mechanism^24^. A similar study reported production of heteropolymeric EPS from monomers (glucose, galactose and mannose) in *Methylobacillus* sp. strain 12S and identified homologous genes (wzx, wzc, wzy and gts) involved in synthesis via Wzy-dependent system^25^. The UDP-GlcNAc epimerase (gne; convert UDP-GlcNAc to UDP-GalNAc) and phosphogluconate dehydrogenase (gnd) genes identified in this strain have also been reported from O-antigen gene cluster of *Escherichia coli*^26^.

The genes for the biosynthesis of LPS core in *Enterobacteriaceae* such as *E. coli*, *Salmonella enteric, S. marcescens* and *Klebsiella pneumoniae* were clustered as ‘waa’ gene cluster on the chromosome. Coderch *et al*.^27^ identified ADP-D-*glycero*-D-*manno*-heptose epimerase (GmhD), ADP-heptose-LPS heptosyltransferase II (WaaF), ADP-heptose-LPS heptosyltransferase I (WaaC), ADP-heptose-LPS heptosyltransferase III (WaaQ) and 3-deoxy-D-manno-octulosonic acid transferase (WaaA) in *S. marcescens* N28b through complementation analysis. *Serratia* sp. ISTD04 showed the presence of LPS core biosynthesis gene cluster (Waa) and also showed the presence of above five genes in the cluster. The two O-antigen genes wecA (undecaprenyl-phosphate alpha-N-acetylglucosaminyl 1-phosphate transferase) and wzx (O-antigen translocase) identified in the cluster are known for O antigen synthesis. Complementation study in *E. coli* K-12 with *E. coli* O7 and *S. enterica* O antigen translocase indicated that complete antigen subunit is not required for translocation and recognition of the Und-P-P-linked (undecaprenylpyrophosphate-linked) sugars by wzx complex is involved in O-antigen processing during LPS synthesis^23^. A Comparative large-scale gene trait-matching approach was applied in 20 sequenced *Bifidobacterium breve* strain to distinguish between EPS producer and non-producers^22^. The EPS-producing cluster was shown to contain multiple glycosyltransferases, priming GTs, flippase, tyrosine kinase, chain length determination proteins and acetyltransferse^22^. Notably these genes are also present in most of the identified clusters of this strain. The diverse monomer composition and the repeating unit of polymer are directly influenced by the specific glycosyltransferase genes in polysaccharide. The impact of different sugar sources (glucose, galactose, lactose and fructose) on EPS biosynthetic gene expression (galE, galU, galT and rmlA-D) was demonstrated for nucleotide sugar production and EPS synthesis in *longum* CRC 002^28^. The GumL (pyruvyltransferase) present in cluster 7 add non-sugar moiety to mannose residues of the polysaccharide repeating unit.

The EPS biosynthesis regulation is a complex process that is regulated at various levels within the microorganisms. The role of kinases in regulation EPS production is well known and it regulates the EPS biosynthetic enzymes through phosphorylation. The two tyrosine-protein kinase (TK) gene known to be involved in regulation of EPS was discovered in cluster 2 and 7. Elsholz *et al*.^29^ reported that EPS acts as a signaling molecule for the TK protein in *Bacillus subtilis*. The TK have two components, one membranous (epsA) and other kinase (epsB). The EPS production is subjected to the positive feedback loop and regulated by EPS concentration. The kinase remains inactivated in absence of EPS by autophosphorylation but the presence of EPS inhibits autophosphorylation and promotes phosphorylation of glycosyltransferase, as a result, stimulating EPS production^29^. Wzc (component of translocation complex) is also known to be regulated by phosphorylation as it contains cytosolic tyrosine autokinase domain.

Diguanylate cyclases synthesize bis-(3′-5′)-cyclic dimeric guanosine monophosphate (c-di-GMP) and this c-di-GMP act as second messenger involved in decision ‘to swim or to stick’. The c-di-GMP level positively stimulates expression and secretion of various EPS^16^. A two-component regulatory system (NarX and NarL) was observed in the cluster and is reported to regulate nitrate reductase (nar GHJI) in EPS-producing halophile *Halomonas maura*^30^. NarX a sensor histidine kinase sense nitrate and activates NarL, the activated NarL recognizes the regulatory regions in the sequence of various other genes and regulates their expression. The nitrogen limitation induces EPS production^15^. Stewartan is high molecular weight, acidic component having repeating units of oligosaccharides. The presence of LuxR family transcriptional regulator is shown to regulate the stewartan synthesis and presence of LuxR family DNA-binding response regulator (NarL) in cluster 2 may be regulating the production of Stewartan. Nitrogen plays an important role in the regulation of EPS production. The induction of EPS production under nitrogen limitation was observed for several strains by regulation nitrogen metabolic genes. Transcriptomic profile of *Agrobacterium* sp. ATCC 31749 under nitrogen starvation showed diguanylate cyclases (c-di-GMP) mediated regulation of EPS synthesis and deletion of this gene resulted in drastic reduction in EPS yield^31^. c-di-GMP mediated regulation of EPS such as cellulose, xanthan and alginate is also reported^15^. Dalsing and Allen.^32^, reported that nitrate reductase in *R. solanacearum* is important for EPS production and also quantitatively proved the role of nitrogen assimilation on EPS production.

### Production EPS by *Serratia* sp. ISTD04 and its morphological observation

The *Serratia* sp. ISTD04 was previously characterized comprehensively for CO_2_-sequestration and accumulation of value-added products^3,5,9^. In this study, we investigated the exopolysaccharide (both loosely bound (LB) and tightly bound (TB)) EPS production by *Serratia* sp. ISTD04 while growing on NaHCO_3_, glucose and NaHCO_3_ plus glucose in mineral medium. The EPS production was found to be 0.88 ± 0.08, 1.25 ± 0.13 and 1.44 ± 0.10 g L^−1^ on glucose, NaHCO_3_ and NaHCO_3_ plus glucose respectively at pH 7.8 **(**Table 1**)**. It was observed from the findings that both glucose and NaHCO_3_ together in the media was required for EPS production. The EPS production was lower in glucose compared to NaHCO_3_ and it was also observed that using glucose as an inducer with NaHCO_3_ was found to be important for EPS production. During initial growth phase, the substrate is present in surplus and the cells are actively involved in growth. But with time as the substrate (carbon and phosphorous) imbalance occurs, the cellular machinery actively engage in EPS production under stress and also changes in cellular morphology and shape can be observed^11^. The EPS production is influenced by several factors such as bacterial growth, pH, temperature, carbon and nitrogen source and their ratio^11,12^. The pH of media is known to significantly influence the EPS production. The microorganisms have been reported to utilize diverse carbon and nitrogen sources for EPS production^33^. C/N ratio plays a critical role in EPS production and any increase or decrease in ratio affect the chemical nature of EPS and its flocculating properties^11^. The production of EPS was further optimized with different concentration of NaHCO_3_ plus glucose, C/N ratios and pH (Table 1). The maximum EPS yield was found to be 4.57 ± 0.27 g L^−1^ at glucose plus NaHCO_3_ (1 g L^−1^ and 50 mM), C/N ratio 17 and pH 8. The EPS production increased almost three fold after optimization of media components. The EPS production has been reported from diverse microorganisms while growing on different carbon source. In a similar report, *Serratia* sp. 1 was shown to produce 3.44 g L^−1^ EPS while growing on wastewater sludge^11^. *Halomonas* species was reported to produce 1.073 g L^−1^ EPS on sucrose^34^. EPS produced by *P. polymyxa* SQR-21(3.44 g L^−1^) on galactose, *Bacillus* sp. ISTVK1 (0.31 g L^−1^) on waste water and sucrose, *Proteus mirabilis* TJ-1 (1.3 g L^−1^) on glucose, *Streptococcus thermophilus* ASCC 1275 (1 g L^−1^) in milk, *Klebsiella* sp.(1.0 g L^−1^) from glucose and *P. jamilae* CP-38 (4.2 g L^−1^) on olive mill wastewater^12,35,36^. Compared to earlier reports, this strain produced a good amount of EPS and that too using CO_2_ and glucose as carbon source. To the best of our knowledge, this is the first report on CO_2_ sequestration and EPS production by bacteria.

**Table 1:** Optimization of process parameters for production of EPS by *Serratia* sp. ISTD04.

Serial No.	Glucose% (w/v)	NaHCO_3_ (mM)	C:N	pH	LB EPS (g L^−1^)	TB EPS (g L^−1^)	EPS (g L^−1^)
1	—	50	3	7.8	1.13 ± 0.11	0.12 ± 0.02	1.25 ± 0.13
2	0.5	50	10	7.8	1.33 ± 0.08	0.11 ± 0.02	1.44 ± 0.10
3	1	50	17	7.8	3.41 ± 0.13	0.24 ± 0.05	3.65 ± 0.18
4	1.5	50	25	7.8	2.95 ± 0.11	0.18 ± 0.03	3.13 ± 0.14
5	2	50	32	7.8	2.83 ± 0.15	0.19 ± 0.02	3.02 ± 0.17
6	2.5	50	40	7.8	2.64 ± 0.09	0.21 ± 0.04	2.85 ± 0.13
7	1	—	14	7.8	0.82 ± 0.07	0.06 ± 0.01	0.88 ± 0.08
8	1	20	15	7.8	3.11 ± 0.10	0.38 ± 0.06	3.39 ± 0.16
9	1	50	17	7.8	3.31 ± 0.14	0.28 ± 0.03	3.59 ± 0.17
10	1	100	20	7.8	2.98 ± 0.17	0.31 ± 0.05	3.29 ± 0.22
11	1	150	21	7.8	2.12 ± 0.14	0.14 ± 0.03	2.26 ± 0.17
12	1	200	24	7.8	1.97 ± 0.10	0. 17 ± 0.02	2.14 ± 0.12
13	1	50	17	5.0	1.86 ± 0.13	0.15 ± 0.04	2.01 ± 0.17
14	1	50	17	6.0	2.31 ± 0.14	0.28 ± 0.03	2.59 ± 0.17
15	1	50	17	7.0	3.47 ± 0.18	0.37 ± 0.04	3.84 ± 0.22
16	1	50	17	8.0	4.18 ± 0.15	0.39 ± 0.06	4.57 ± 0.21
17	1	50	17	9.0	3.53 ± 0.17	0.33 ± 0.04	3.88 ± 0.21
18	1	50	17	10	2.52 ± 0.16	0.34 ± 0.03	2.86 ± 0.19

**Figure 3:**
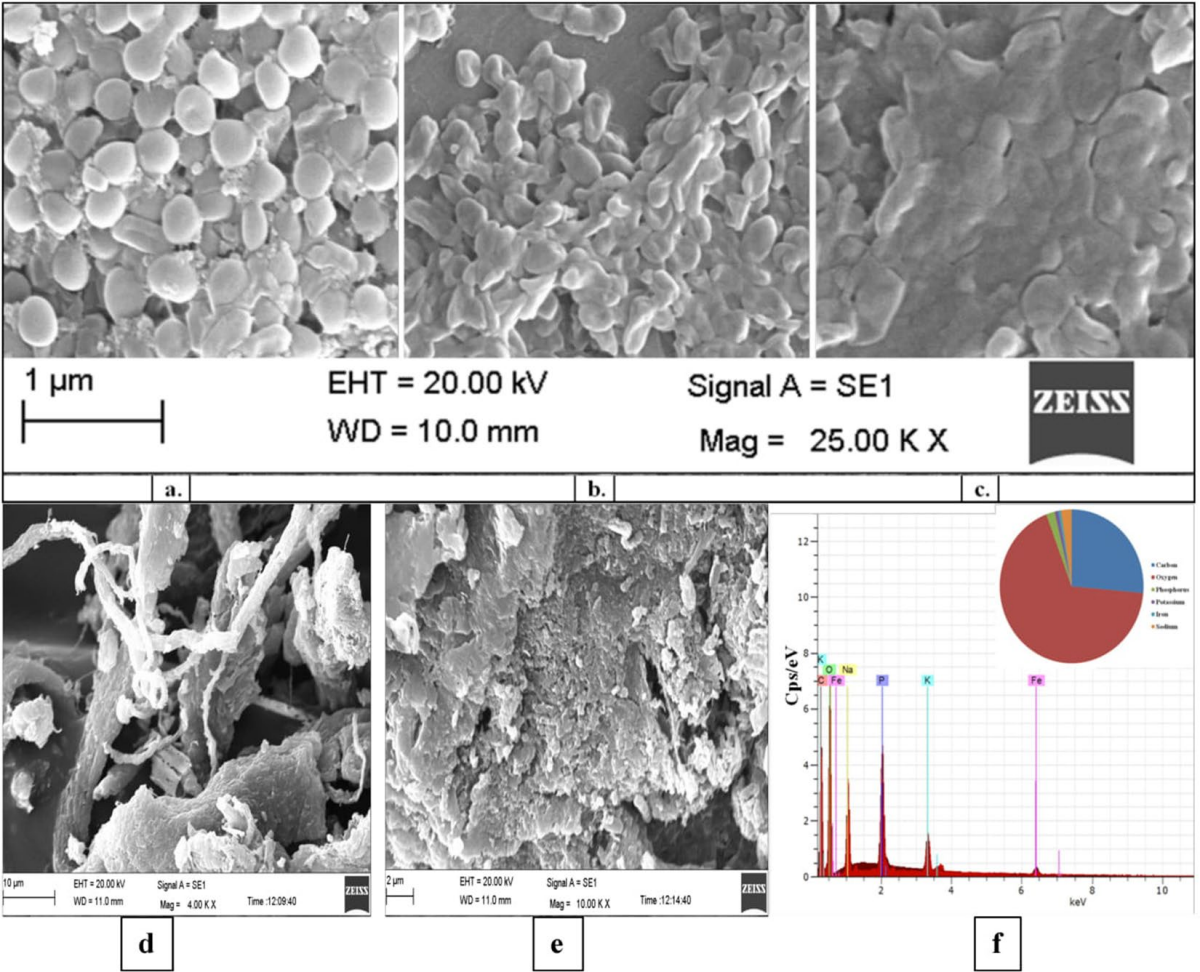
(**a**) SEM observation of *Serratia* sp. ISTD04 in MM-media supplemented with 0.5% glucose (w/v) and 50 mM NaHCO3 at **(a)** 0 h **(b)** 24 h **(c)** 72 h. SEM image of purified EPS extracted from *Serratia* sp. ISTD04 at various magnifications (**d**,**e**). Elemental composition of EPS detected by EDX **(f)** peaks of the elements detected and their abundance (pie chart).

The SEM analysis of bacterial culture infers that EPS production was minimal initially but with time a mat of EPS can be seen and it’s no longer possible to recognize the individual bacterial cells after 72 h of growth (Fig. 3a–c). The extracted EPS appears as a white thread like granular material (indicative of gelling and emulsifying properties) as evident from the Fig. 3d,e. Similar morphology was observed for *R. mucilaginosa* UANL-001L EPS^14^. The microbes isolated from extreme habitats have an innate feature to produce EPS especially to get a hold on the rocky surface. This bacterium was isolated from marble mining rocks so EPS production can be viewed as an intrinsic quality of the microorganisms inhabiting such a diverse ecological niche.

### Characterization of EPS

#### Estimation of carbohydrate, lipid and proteins present in EPS

The total carbohydrate content and reducing sugar was estimated for extracted, purified and dried EPS from *Serratia* sp. ISTD04. The total carbohydrate (mono-, di-, oligo-) present in EPS was quantified by phenol-sulfuric acid and it was found to be 67% w/w of total EPS. The amount of reducing sugar present in EPS was estimated by 3, 5-dinitrosalicylic acid (DNS) method. The reducing sugar was found to be 34.5% w/w. The result confirms that carbohydrates are the major component of the EPS. The total carbohydrate and reducing sugar estimation revealed the EPS produced by *Serratia* sp. ISTD04 contains an almost equal representation of reducing and no-reducing sugar. The total protein of EPS was estimated by Bradford assay and the protein content was found to be 0.54% w/w. The lipid content of EPS was estimated by the process described earlier^37^. The lipid content was found to be 10.49% w/w of dry EPS. The probable reason for high lipid content may be because this bacterium has already been reported for production of extracellular fatty acids^2^. According to earlier reports, the lipid content of EPS varies from 1–10%. The lipid, protein and their composition present in the EPS plays an important role in determining the properties (such as flocculation, adsorption, transformation of chemicals and metal binding) of EPS^12,33,37^.

### Characterization of EPS for functional group composition and linkage analysis

#### SEM-EDX analysis of EPS

The surface texture and composition of polysaccharide was analyzed using SEM-EDX at various magnifications (Fig. 3f). The polysaccharide appears to be an amorphous white solid under low magnification and the higher magnifications revealed a porous, layered texture of EPS, with pore sizes varying in the range from nanometers to micrometers. EDX analysis of the purified EPS revealed the dominance of carbon and oxygen along with the presence of other elements like sodium, iron, phosphorus, potassium in trace amount (Fig. 3f). The EDX analysis confirmed the organic nature of EPS.

#### Functional group analysis by FT-IR

The purified and dried EPS of *Serratia* sp. ISTD04 was characterized for functional group composition by FT-IR and the bands were assigned based on previous literature (Fig. S1). The broad and intense band at 3396.63 cm^−1^ and 2929.86 cm^−1^ represents stretching vibration of hydroxyl groups and C-H stretching of CH_2_ groups respectively of polysaccharide^36^. The band at 1643.34 cm^−1^ (C=O stretching vibration; flexural vibration peak of O-H), 1537.26 cm^−1^ (vibration of C-O), 1414.81 cm^−1^ (CH_2_ bending and C-O-O stretching vibrations), 1390.67 cm^−1^ (bending vibration of C-H) indicate the presence of amide, acetate and carboxylate group^38^. The strong absorption band in the range 1200–1000 cm^−1^ represents anomeric region C-O-C and C-O stretching of polysaccharide^36^. The band at 1078.20 cm^−1^ is characteristic for the presence of β -glucans due to O-substituted glucose residues^39^. The band at 854.46 cm^−1^ (C-H variable angle vibration) indicated the presence of β-pyranoside and mannopyranoside units^40^. The absorption band at 707.87 and 543.92 cm^−1^ can be detected in (1 → 3)-β-D-glucan^40^. The FIR spectra analysis indicated the presence of the characteristic bands of EPS.

#### Monosaccharide composition and linkage analysis by GC-MS

The monosaccharide components present in EPS produced by *Serratia* sp. ISTD04 were analyzed by GC–MS after hydrolysis and silylation^36,40^. The GC-MS total ion chromatogram (TIC) has been shown in Fig. S2(a). In TIC, the fragmentation pattern of mass spectra detected three types of monosaccharide i.e., glucose, galactose, mannose and N-Acetylglucosamine (GlcNac) derivative in the EPS. The monosaccharide α-D-glucose and hexopyranoside (galactosidase) was detected at R.T. (Retention Time) 16. 28, 16.60 and 43.101. β- GlcNac derivative (R.T. 14.890), β-D-mannofuranose (R.T. 20.703 and 23.41) and all the detected monosaccharide showed D-configuration. Moreover, these results validated the prominent bands of functional groups detected in the FT-IR spectrum. The linkage analysis gives significant information about the bonding pattern of monosaccharide in the EPS. The linkage analysis of EPS was determined by pre-methylation and then hydrolysis as described^40^. The TIC and fragmentation pattern of Mass spectra revealed the presence of β-D-glucopyranoside, methyl 2,4,6-tri-O-methyl- (R.T. 18.467), which corresponded to 3-linked glucose residue **(**Fig. S2(b)**)**^36^. Another peak at R.T. 24.917 and 27.367 represent the 2, 3, 4, 5- tetra-O-methyl arising from the terminal branched glucose and mannose residues. The monomer composition and linkage analysis indicated that the EPS is heteropolymer with diverse linkages.

#### Structural configuration Analysis of EPS by ^13^C and ^1^H NMR

The ^1^H NMR predicts the glycosidic bond configuration of the polysaccharide as shown in Fig. S3(a). The anomeric region resonance signal occurs in the range (4.5–5.5 ppm) of sugar molecule in polysaccharide. The two major anomeric region (carbon-containing proton) resonance signal was observed at 4.6 and 5.1 ppm. The other different C2 to C6 proton signal of the glycosidic ring was observed between 3.13–3.73 ppm^41^. The ^13^C spectra also supported the ^1^H findings and pyranose ring signal between (60–80 ppm) i.e., 75.96, 74.16, 75.78, 69.67 and 60.61 ppm representative of C3, C5, C2, C4 and C6 was observed in the chromatogram **(**Fig. S3(b)**)**. The absence of resonance signal between 82–88 ppm indicate presence of sugar residues only in the form of pyranoid glycosides^38^. The two anomeric carbon region resonance signals were observed at 92.11 and 95.93 ppm and with predominant alpha linkage. The signal at 60–61 ppm and downfield shift at 69.62 ppm is the characteristic of glucose moiety in the backbone structure of EPS^34,41^. In the ^13^C NMR spectra, the secondary alcohol of carbohydrates and glycosidic carbon of polysaccharide resonates in between ~60–90 ppm and ~95–106 ppm respectively. The glycosidic carbon also indicates whether the carbon is attached to alpha oxygen (95–103 ppm) or beta oxygen (103–106 ppm) in linkage^42^. The ^1^H and ^13^C resonance signal of proton and carbon of sugar moieties indicated the presence of more than one sugar residues with predominant alpha oxygen linkage in the polysaccharide.

### Application of EPS produced by *Serratia* sp. ISTD04

#### Determination of the flocculating activity of EPS

The polysaccharides are well known for their flocculating properties. The bioflocculation characteristic of EPS was evaluated by kaolin test (Jar test). The results show that the flocculating activity of bacterial culture broth EPS and supernatant EPS was 68% ± 0.9 and 59% ± 0.6 respectively. A higher flocculating activity of culture broth EPS as compared to supernatant EPS may be due to more protein content in the broth EPS as it is composed of LB-EPS and TB-EPS^26^. As compared to previous literature the flocculating activity of EPS is in the range of 61–95% using purified EPS as well as direct bacterial culture broths^11,43^. Application of purified EPS as flocculating agent may be not a cost-effective technique, because major cost involves in centrifugation, precipitation and purification of EPS put some extra cost on its application as bioflocculant. Using direct culture broth may be a sustainable approach to reduce the cost involved in the application of EPS as bioflocculant.

#### Determination of dyes decolorization by EPS

**Figure 4:**
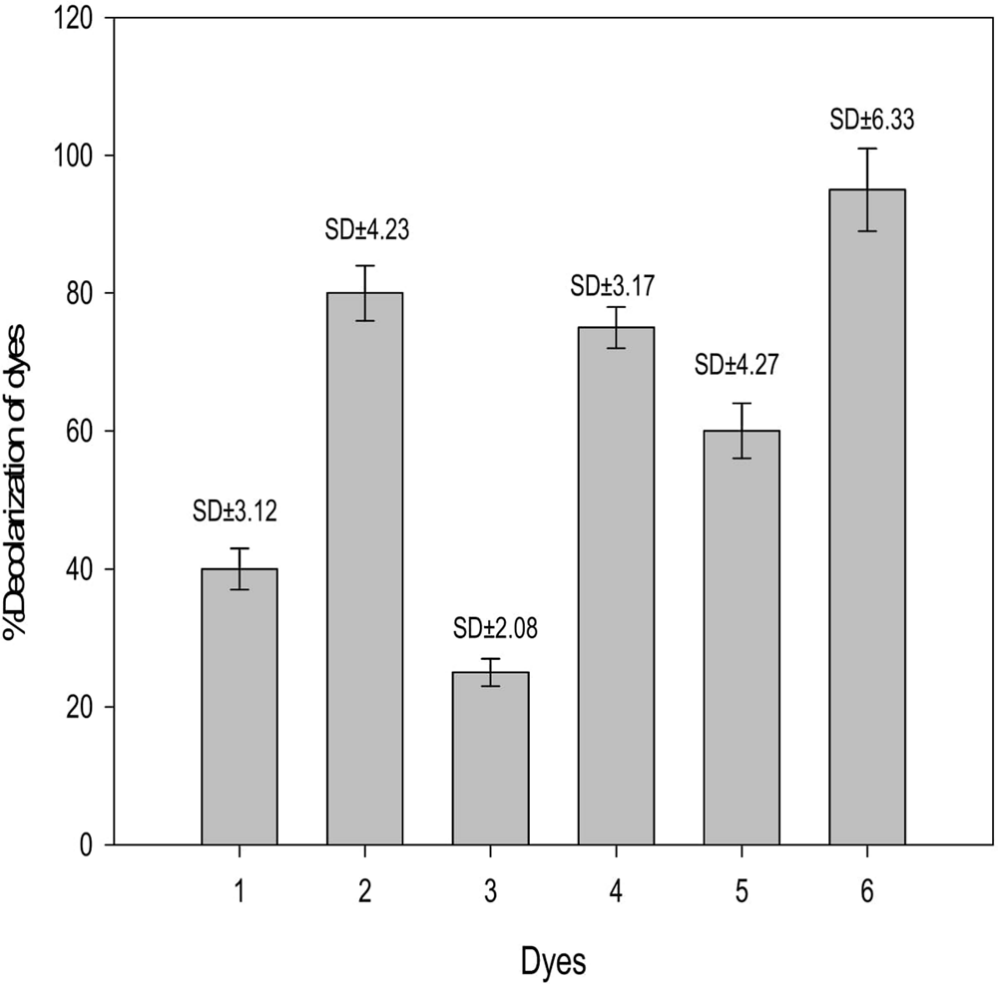
Representation of dyes decolorization efficiency of EPS produced by *Serratia* sp. ISTD04 (1) Trypan blue (2) Acridine orange (3) Methyl orange (4) Bromothymol blue (5) Aniline blue (6) Crystal violet and error bar represented as standard deviation (SD).

There are various physical, chemical and biological dyes decolorization technologies previously described by the researcher, such as biodegradation, sorption, ozonolysis and precipitation^44^. Biosorption is considered as a promising technology due to low cost, high-removal efficiency, and less labor-intensive operation for the removal of dyes from industrial effluents and natural waters^44^. Some agricultural byproducts (rice husk, bark and orange peel) and microorganisms (bacteria, fungi and algae) and their materials have been used as bioflocculants to remove dyes^45^. EPS produced by *Serratia* sp. ISTD04 was used as bioflocculant for decolorization of dyes. Six dyes were selected, four anionic dyes (trypan blue, methyl orange, bromothymol blue, aniline blue) and two cationic dyes (acridine orange, crystal violet) at a dye concentration 0.1% and pH 7. The flocculant effectively decolorized the anionic dye, such as trypan blue (40%), methyl orange (25%), bromothymol blue (75%) and aniline blue (60%) (Fig. 4). The decolorization activity of the bioflocculant was much more effective against the cationic dye, such as acridine orange (80%) and crystal violet (95%) and this might be due to anionic nature of the bioflocculant (EPS). Biopolymer mediated flocculation occurs through bridging and charge neutralization mechanism. The efficacy of the bridging mechanism depends on the molecular weight and charge on the polymer, the ionic strength of suspension, and the nature of mixing^12^. These results shows the cost-effective production of EPS by chemolithotrophic bacteria *Serratia* sp. ISTD04 and its application as bioflocculant for decolorization of anionic dyes.

## Methods

### Chemicals and reagents

All chemicals used in the present study were obtained from Sigma–Aldrich (St. Louis, MO, USA) or Merck (Darmstadt, Germany) or HiMedia (India). All the organic solvents used were of analytical reagent grade.

### Genomic analysis of *Serratia* sp. ISTD04

The *Serratia* sp. ISTD04 has a genome size of 5.07 Mb and coverage of 81X was achieved. This whole-genome shotgun project has already been deposited at DDBJ/ENA/GenBank under the accession number MBDW00000000.1 and available online. The high-quality reads were filtered, genes were annotated and pathways were predicted as described earlier^17^. An assessment of genes of *Serratia* sp. strain ISTD04 with respect to its genome was carried out with the help of clicO FS (Circular Layout Interactive Converter Free Services). The gene annotation, pathways and cluster analysis of *Serratia* sp. ISTD04 genome was also performed by Rapid Annotations using Subsystems Technology (RAST) and antiSMASH ver. 4.1.0^46,47^.

### Microorganism and culture condition

An earlier reported carbon concentrating bacterial strain *Serratia* sp. ISTD04 (gene bank accession number- JF276275) was used for CO_2_ sequestration along with EPS production. Pre-LB cultured of the strain was transferred (1:10 v/v) to mineral medium (MM) with slight modification containing (g L^−1^): Na_2_HPO_4_ 5.0, KH_2_PO_4_ 6.0, ammonium ferric citrate 2.0, MgSO_4_ 1.0, CaCl_2_ 0.05, yeast extract 0.5, trace elements solution 1 mL, consisting of (g L^−1^): FeSO_4_.7H_2_O 5.0, MnSO_4_ 2.0, CoCl_2_ 1.0, ZnCl_2_ 1.0, dissolved in 0.1 N HCl solution pH 7.8 and supplemented with filter sterilized 50 mM NaHCO_3_ and 5 g L^−1^ glucose as carbon sources and incubated under aerobic conditions at 30 °C and 150 rpm for several days^41^. The optimization experiments were performed with *Serratia* sp. ISTD04, pre-cultured in LB and inoculated in 250 mL Erlenmeyer flasks under aerobic conditions, with 100 mL of MM at 30 °C and 150 rpm for 72 h and was supplemented with glucose concentration (0.0–2.5% w/v), NaHCO3 concentration (0–200 mM) and pH ranges (5–10) as per experimental design, based on the previous studies carried out on the bacterial strain^9^. C/N ratio play a crucial role in production of EPS by the microorganism^12^, so in this study, C/N ratio (3–40) was also optimized. Finally, the weight of EPS was determined after its purification and expressed in g L^−1^ ^11,34,41^ followed by its detail characterization discussed below.

### Morphological observation of *Serratia* sp. ISTD04 by Scanning Electron Microscopy (SEM)

For SEM analysis, Cells were harvested from 0 h, 24 h and 72 h culture and fixed with glutaraldehyde (1% solution) and paraformaldehyde (2%) buffered with sodium phosphate buffer saline (0.1 M, pH 6.8) for 12–18 h at 4 °C. The cells were coated by Sc 7640 sputter coater (VG Microtech, East Sussex, TN22, England) for 30 min further processed as described earlier^48^. The coated cells were viewed at 15 kV with SEM (Model-Zeiss EVO40, Germany).

### Isolation, extraction and purification of EPS

The cultures were harvested and centrifuged at 10000 rpm for 15 min and the supernatant and pellets were processed for EPS extraction. The EPS from culture supernatant was extracted by adding equal volumes of ice-cold isopropanol with proper mixing followed by overnight storage at 4 °C. The capsular EPS was extracted from the pellet as described^11^. The precipitated EPS from supernatant and pellet was concentrated by cold centrifugation at 4 °C for 30 min at 15,000 rpm. The EPS pellet was washed thrice with ice-cold isopropanol and acetone to remove impurities. The purified EPS was then vacuum dried till a constant weight achieved and then weighed (g L^−1^)^34,41^.

### Chemical characterization of EPS

#### Estimation of total carbohydrate, reducing sugar, protein and lipid content of EPS

The major composition of EPS is carbohydrate but it also contains some non-carbohydrate component. The total carbohydrate content and reducing sugar content was estimated by Phenol sulphuric acid (PS) method and dinitrosalicylic acid (DNS) method using glucose as a standard^39,49^. Briefly, for PS, 500 µL of 80% phenol solution was added to 100 µL sample (EPS 2.5 mg mL^−1^) followed by vortexing and addition of 2 mL H_2_SO_4_. The solution was allowed to stand for 10 min at room temperature (25 °C) and the optical density (O.D.) was taken at 490 nm against blank. For DNS 500 µL of DNS was added to 500 µL of sample (EPS 1 mg mL^−1^) and boiled for 10 min. on water bath followed by addition of 150 µL sodium potassium tartarate (40%) and allowed to cool at room temperature followed by absorbance at 575 nm with respect to blank. The total protein content of EPS was quantified by Bradford assay^50^. 900 µL of Bradford reagent was added to 100 µL sample (EPS 5 mg mL^−1^) mixed well and incubated in the dark for 30 min at room temperature. The absorbance was taken at 595 nm. Total lipid content of EPS was measured by chloroform and methanol (2:1) extraction^37^. The lipid content in the ESP was expressed in percentage.

#### Structural composition analysis of EPS

Surface structure, composition and bonding patterns of the extracted and dried EPS was characterized by Scanning Electron Microscopy-Energy Dispersive X-ray (SEM-EDX) spectroscopy, Fourier Transform Infrared Spectroscopy (FTIR), Gas Chromatography-Mass Spectrometry (GC-MS) and Nuclear Magnetic Resonance (NMR) analysis.

#### SEM-EDX analysis of EPS

SEM was performed for surface texture analysis of the purified dried EPS produced from *Serratia* sp. ISTD04. The dried EPS powder was mounted and coated as described earlier^48^ mounted on aluminum stubs and the sample was coated with 90 Å thick gold-palladium coating in polaron Sc 7640 sputter coater (Carl Zeis, Germany) for 30 min. Coated samples were viewed at 20 KV with scanning electron microscopy (Leo Electron Microscopy Ltd., Cambridge). Energy Dispersive X-ray (EDX) spectroscopy of purified EPS was done for qualitative analysis of the elemental composition of EPS. The analysis was performed at 20 kV on Dx4 Prime EDX spectrometer (Bruker, Germany) equipped with X-flash detector.

#### FT-IR analysis

Functional groups present in EPS was characterized by Fourier Transform Infrared Spectroscopy (FTIR). For FT-IR analysis, dried EPS was properly mixed with potassium bromide (KBr) and compressed to prepare a disc of about 3 mm in diameter. IR spectroscopy of the disc was recorded Varian 7000 FTIR spectrometer (Perkin-Elmer Inc., Wellesley, MA, USA) at room temperature, in the frequency range of 400 to 4000 cm^−1^, 64 scans per sample at a resolution of 4 cm^−1^.

#### GC-MS analysis

The monosaccharide composition and linkage analysis of EPS was determined by GC-MS analysis. The monosaccharide composition was analyzed by hydrolysis and silylation of dried and powered EPS to obtain volatile derivatives (methylsilanes) for GC-MS analysis^36,40^. In brief, 5–10 mg of EPS was hydrolyzed with Trifluoroacetic acid (1 M) at 100 °C for 8 h followed by reduction with 100 µL NaBH_4_ (10 mg in 1 mL of 1 M ammonium hydroxide) for 16 h at room temperature after proper mixing. The reduced sample was silylated with N, O-Bis (trimethylsilyl) trifluoroacetamide (BSTFA) in pyridine at 60 °C for 30 min.

Linkages analysis of EPS was determined by permethylation followed by hydrolysis and silylation^34,40^. 5–10 mg of EPS sample suspended in 1 mL dimethyl sulphoxide (DMSO) was mixed with sodium hydroxide (50 mg) and 0.1 mL of methyl iodide. The samples were kept on a shaker at room temperature for 5 h followed by organic extraction of the aqueous phase with dichloromethane. The organic layer was water washed and evaporated to dryness under an air stream. The methylated product was further hydrolyzed and silylated as described above. The GC-MS analysis of both permethylated and silylated EPS in pyridine was performed by condition described^36^ on Shimadzu GC-MS-QP 2010 Plus instrument equipped with a capillary column Rtx-5 (dimensions: 0.25-μm film thickness, 0.25 mm ID, 30 m in length). All data was matched with the GC-MS inbuilt standard mass spectra library of NIST-08 and Wiley-8.

#### NMR analysis

The ^1^H NMR and ^13^C NMR measurements of pure EPS was performed using Varian Mercury Plus NMR spectrometer equipped with ATB and SW Varian probes (5 mm). Purified and dried EPS (10 mg mL^−1^) was dissolved in deuterated water^41^. ^1^H spectrum was recorded at 10330.578 Hz, with a pulse width of 3.17 s, pulse duration of 64° and a recycle delay of 1 s. The spectrum was measured with 16 scans. ^13^C NMR spectra was obtained at 29761.904 Hz, with a pulse width of 1.10 s, pulse duration of 64° and a recycle delay of 0.03 s. The spectrum was measured with 1640 scans.

### Application of EPS

#### Determination of flocculating activity

The flocculation activity of EPS was tested by kaolin flocculating method. The mixture contained 100 ml kaolin clay suspension (5 g L^−1^, pH 7.0), 0.2 ml EPS and 1 ml CaCl_2_ solution (1 mg L^−1^, pH 7.0), stirred vigorously and left standing for 10 min. The absorbance of the supernatant was measured at 550 nm with respect to control and the flocculating activity was calculated^43^.

#### Application of EPS in dyes decolorization

The decolorization activity of EPS on different dyes was carried out. A 0.1% w/v of cationic dye (Acridine orange, Crystal violet) and anionic dye (Trypan blue, Methyl orange, Bromothymol blue, Aniline blue) was incubated with 1.0 ml culture supernatant and 1 ml CaCl_2_ solution (1 mg L^−1^) at room temperature. After the addition of bioflocculants, the compound in the beaker was mixed using a blender at 200 rpm for 1 min, and then at 40 rpm for another 3 min. The dyes solution was left to settle for 10 min, and then the supernatant was taken for analysis^43^. The decolorization of each dye was measured using a UV–vis spectrophotometer in scan mode (200–800 nm) at their maximum wavelength i.e., 607, 470–490, 407, 615, 588, 595–610 nm for trypan blue, acridine orange, methyl orange, bromothymol blue, crystal violet and aniline blue, respectively. The residual concentration of the dye in the samples was then calculated, and the decolorization efficiency was calculated^43^.

## Conclusion

Rising level of atmospheric CO_2_ could be mitigated by bacterial fixation of CO_2_ along with production of value added product. The genomic investigation of previously reported CO_2_ sequestrating bacterial strain *Serratia* sp. ISTD04 discovered the presence of several contender genes which are responsible for CO_2_ sequestration and production of EPS. Nr blast analysis of genome of *Serratia* sp. ISTD04 also supported the findings. Among the 13 enzymes of CBB cycle, 12 enzymes are annotated successfully in the genome including key enzyme PRK. In addition to CO_2_ this strain can also metabolize monosaccharide, disaccharides, polysaccharides and many organic compounds as carbon source. The gene cluster and enzymes responsible for production of EPS were also predicted in the genome of the strain. The production of EPS and its optimization revealed that, the strain can produce EPS up to 4.57 ± 0.21 g/L using NaHCO_3_ along with glucose as a carbon source. Characterization of EPS was performed by various techniques and it confirmed the presence of sugar as the major component along with lipids and protein. Further flocculating activity and dye decolorization efficiency of EPS was evaluated and established the environmental application of EPS as bioflocculent especially against the cationic dye. The present study directed the pathways and enzymes responsible for CO_2_ sequestration along with the production of EPS, may applied in future for biovalorization of CO_2_.

## Supplementary information

Dataset 1
