# Supplementary material for: Genomic analysis of carbon dioxide sequestering bacterium for exopolysaccharides production
Source: Sci Rep. 2019 Mar 12;9:4270. doi: 10.1038/s41598-019-41052-0 (PMC6414628; doi:10.1038/s41598-019-41052-0)
Supplement: Supplementary file 1 — Dataset 1 [file 41598_2019_41052_MOESM1_ESM.doc]

**Supplementary data**

**Title:** **Genomic analysis of carbon dioxide sequestering bacterium for exopolysaccharides production**

**Author names and affiliations:**

Manish Kumar^a^, Madan Kumar^a^, Ashok Pandey^b^ and Indu Shekhar Thakur^a^*

*^a^School of Environmental Sciences, Jawaharlal Nehru University, New Delhi- 110067, India*

*^b^CSIR-Indian Institute of Toxicology Research, 31 MG Marg, Lucknow-226 001, India*

*Corresponding Author


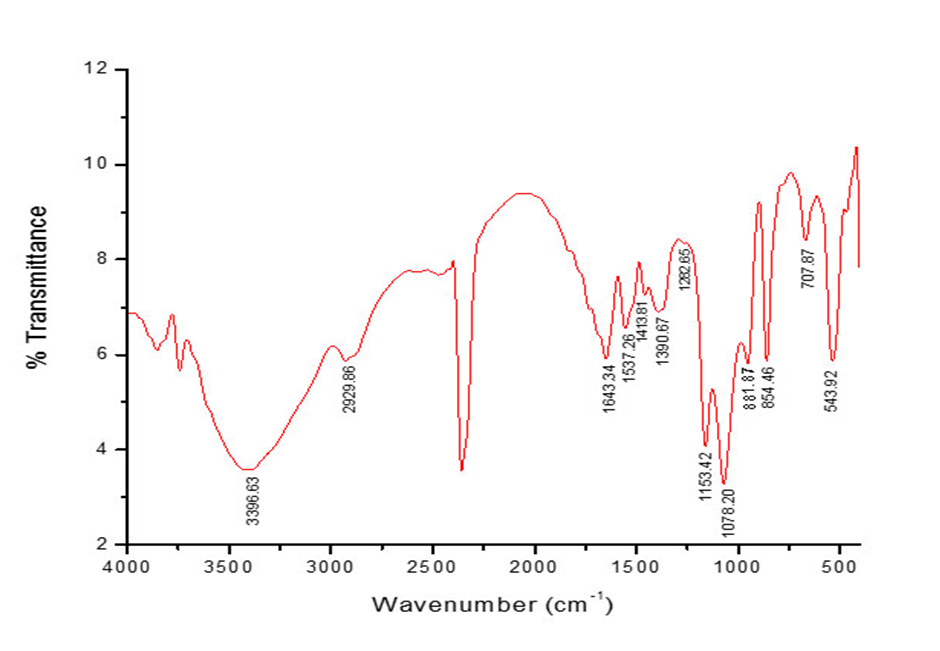
 **Fig. S1** FT-IR spectra of EPS produced by *Serratia* sp. ISTD04.


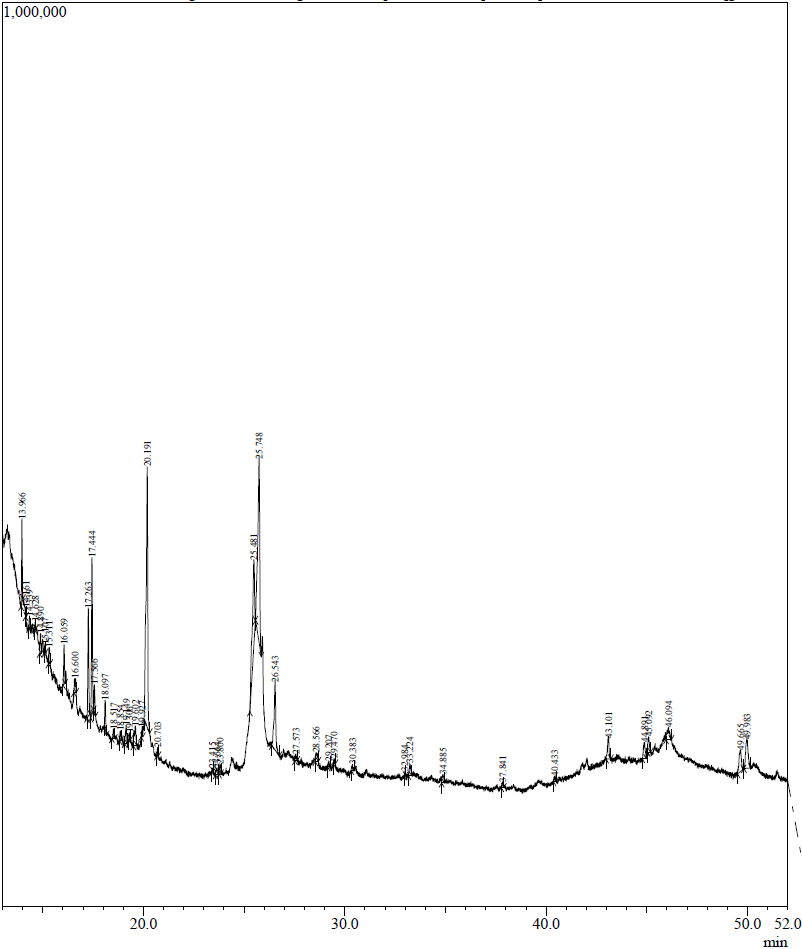


**(a)**


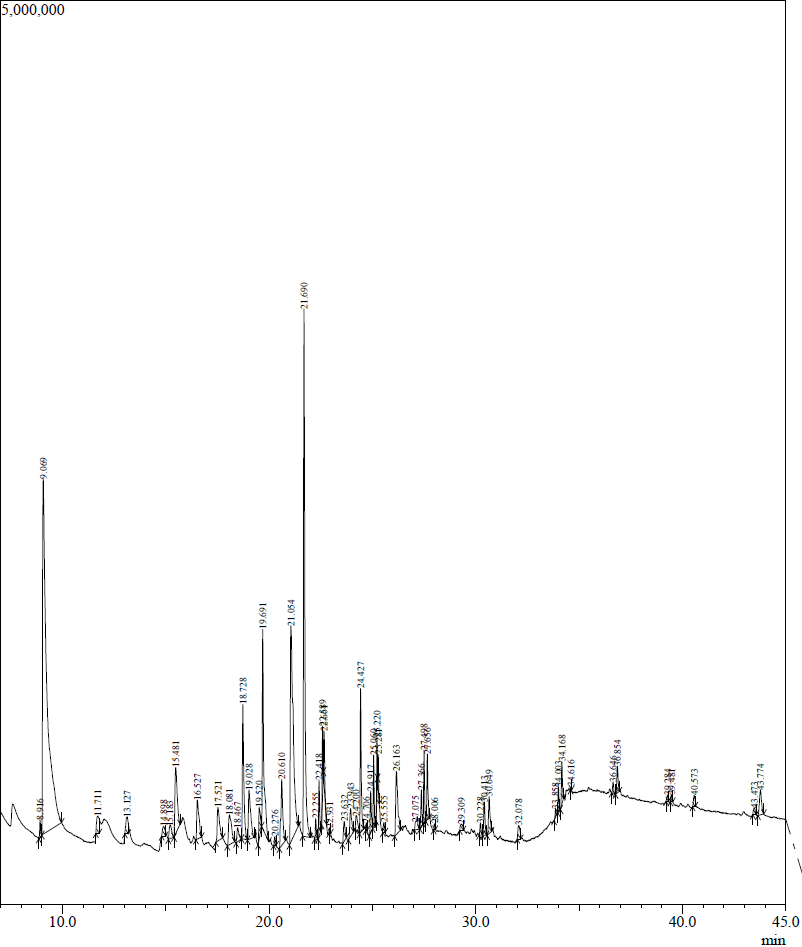


**(b)**

**Fig. S2** **(a)** GC-MS analysis of monomers composition of EPS produced by Serratia sp. ISTD04 **(b)** Linkage analysis of EPS

**(a)**

**(b)**

**Fig. S3** **(a)** ^13^C and **(b)** ^1^H NMR analysis of EPS extracted from *Serratia* sp. ISTD04

**Table S1 Detail of genes in selected pathway represented on circus plot of Serratia sp. ISTD04 genome**

| **Carbon dioxide sequestration pathway related genes** | | | | | |
| --- | --- | --- | --- | --- | --- |
| ***Serratia* sp. ISTD04** | **Name of Enzyme** | **Start** | **End** | **Stand** | **Gene Id** |
| NZ_MBDW01000101.1 | Phosphoribulokinase (PRK) | 39694 | 40563 | - | RS21380 |
| NZ_MBDW01000027.1 | Carbonic anhydrase (can) | 23602 | 24258 | + | RS06440 |
| NZ_MBDW01000046.1 | Phosphoglycerate kinase (PGK) | 15179 | 16342 | + | RS09745 |
| NZ_MBDW01000069.1 | Glyceraldehyde 3-phosphate dehydrogenase (GAPDH) | 84456 | 85451 | - | RS15220 |
| NZ_MBDW01000005.1 | Triosephosphate isomerase (TPI) | 23641 | 24408 | + | RS01160 |
| NZ_MBDW01000046.1 | Fructose-bisphosphate aldolase, class II (FBA) | 16459 | 17538 | + | RS09750 |
| NZ_MBDW01000082.1 | Fructose-bisphosphate aldolase, class I (fbaB) | 6003 | 7052 | - | RS17235 |
| NZ_MBDW01000047.1 | Fructose-1,6-bisphosphatase I (FBP) | 38661 | 39665 | - | RS10880 |
| NZ_MBDW01000005.1 | Fructose-1,6-bisphosphatase II (glpX) | 18250 | 19260 | + | RS01130 |
| NZ_MBDW01000041.1 | Transketolase (tktA, tktB) | 10 | 696 | + | RS09165 |
| NZ_MBDW01000001.1 | D-sedoheptulose 7-phosphate isomerase (lpcA) | 122540 | 123121 | - | RS00600 |
| NZ_MBDW01000101.1 | Ribulose-phosphate 3-epimerase (RPE) | 15631 | 16308 | + | RS21270 |
| NZ_MBDW01000046.1 | Ribose 5-phosphate isomerase A (rpiA) | 27544 | 28200 | + | RS09805 |
| NZ_MBDW01000064.1 | Malate dehydrogenase NADP+ | 58544 | 60823 | - | RS14240 |
| NZ_MBDW01000047.1 | Malate dehydrogenase (mdh) | 43398 | 44336 | + | RS10905 |
| NZ_MBDW01000055.1 | Phosphoenolpyruvate carboxylase (ppc) | 17173 | 18991 | + | RS13175 |
| NZ_MBDW01000013.1 | Phosphoenolpyruvate carboxykinase (ATP) (pckA) | 13332 | 14951 | - | RS03190 |
| **Fatty acid metabolism and PHA biosynthesis pathway related genes** | | | | | |
| NZ_MBDW01000026.1 | Pyruvate water dikinase (ppsA) | 9718 | 12096 | + | RS06255 |
| NZ_MBDW01000083.1 | Fumarate hydratase, class I (fumA, fumB) | 6284 | 7930 | - | RS17330 |
| NZ_MBDW01000081.1 | Fumarate hydratase, class II (fumC) | 70762 | 72159 | + | RS16995 |
| NZ_MBDW01000071.1 | Succinate dehydrogenase / fumarate reductase (sdhA,frdA) | 35940 | 37706 | + | RS15905 |
| NZ_MBDW01000071.1 | Succinyl-CoA synthetase alpha subunit (sucD) | 44090 | 44962 | + | RS15930 |
| NZ_MBDW01000071.1 | Succinyl-CoA synthetase beta subunit (sucC) | 42924 | 44090 | + | RS15925 |
| NZ_MBDW01000038.1 | Acetyl-CoA C-acetyltransferase (atoB) | 14880 | 16064 | - | RS08595 |
| NZ_MBDW01000009.1 | Isocitrate dehydrogenase (IDH1, IDH2) | 30228 | 31481 | - | RS02625 |
| NZ_MBDW01000069.1 | Aconitate hydratase (acnA) | 163283 | 165955 | + | RS15605 |
| NZ_MBDW01000046.1 | Acetyl-CoA carboxylase carboxyl transferase (accA) | 207558 | 208517 | + | RS10595 |
| NZ_MBDW01000030.1 | Methylenetetrahydrofolate dehydrogenase (NADP) (fold) | 59425 | 60291 | + | RS06820 |
| NZ_MBDW01000031.1 | Phosphate acetyltransferase (pta) | 36482 | 38641 | - | RS07200 |
| NZ_MBDW01000031.1 | Acetate kinase (ackA) | 258090 | 258980 | + | RS07205 |
| NZ_MBDW01000015.1 | Acetyl-CoA synthetase (acs) | 1 | 1485 | + | RS03710 |
| NZ_MBDW01000087.1 | Acyl carrier protein (acpP) | 34236 | 34472 | - | RS17880 |
| NZ_MBDW01000087.1 | Acyl-carrier-protein S-malonyltransferase (fabD) | 35373 | 36302 | + | RS17890 |
| NZ_MBDW01000087.1 | 3-oxoacyl-[acyl-carrier-protein] synthase III (fabH) | 36321 | 37274 | - | RS17895 |
| NZ_MBDW01000040.1 | 3-oxoacyl-[acyl-carrier-protein] synthase I] (fabB) | 10451 | 11671 | - | RS09110 |
| NZ_MBDW01000087.1 | 3-oxoacyl-[acyl-carrier-protein] synthase II (fabF) | 32912 | 34153 | - | RS17875 |
| NZ_MBDW01000044.1 | 3-oxoacyl-[acyl-carrier protein] reductase (fabG) | 78612 | 79361 | - | RS09500 |
| NZ_MBDW01000089.1 | 3-hydroxyacyl-[acyl-carrier protein] dehydratase (fabA) | 94540 | 95058 | + | RS18505 |
| NZ_MBDW01000008.1 | Trans-2-enoyl-CoA reductase (NAD+) (fabV) | 6658 | 7857 | + | RS02425 |
| NZ_MBDW01000020.1 | Long-chain acyl-CoA synthetase (fadD) | 36370 | 37908 | + | RS04395 |
| NZ_MBDW01000077.1 | Acetyl-CoA acyltransferase (fadA, fadI) | 38084 | 39386 | + | RS16620 |
| NZ_MBDW01000048.1 | 3-hydroxyacyl-CoA dehydrogenase (fadB) | 40082 | 41008 | - | RS11400 |
| NZ_MBDW01000069.1 | Acetaldehyde dehydrogenase / alcohol dehydrogenase (adhE) | 108698 | 111370 | - | RS15350 |
| **Carbohydrate metabolism and EPS biosynthesis pathway related genes** | | | | | |
| NZ_MBDW01000077.1 | Glucokinase (glk) | 1446 | 2408 | + | RS16415 |
| NZ_MBDW01000071.1 | Phosphoglucomutase (pgm) | 11657 | 13300 | + | RS15785 |
| NZ_MBDW01000089.1 | UTP--glucose-1-phosphate uridylyltransferase (galU, galF) | 306139 | 307032 | - | RS19360 |
| NZ_MBDW01000089.1 | UDP-glucose 4-epimerase (gale) | 276794 | 277807 | - | RS19280 |
| NZ_MBDW01000069.1 | UDPglucose 6-dehydrogenase (ugd) | 104331 | 105674 | + | RS15330 |
| NZ_MBDW01000001.1 | Fructokinase (scrK) | 33986 | 34900 | - | RS00160 |
| NZ_MBDW01000081.1 | Mannose-6-phosphate isomerase (manA) | 68546 | 69724 | - | RS16985 |
| NZ_MBDW01000089.1 | Phosphomannomutase (manB) | 277998 | 279371 | - | RS19285 |
| NZ_MBDW01000089.1 | Mannose-1-phosphate guanylyltransferase (manC) | 279386 | 280810 | - | RS19290 |

**Table S2 Representation of proteins identified against the Nr-blast search in *Serratia* sp.ISTD04 important for CO_2_ sequestration and EPS production**

| **Query locus tag** | **NR_Db_Id (Subject)** | **Description** | **% Identity** | **Alignment length** | **E-value** | **Query cover** |
| --- | --- | --- | --- | --- | --- | --- |
|  |  | **CO2 Sequestering Enzymes** |  |  |  |  |
| BBC05_16825 | CDS56108.1 | phosphoribulokinase [Serratia symbiotica] | 95.156 | 289 | 0 | 100 |
| BBC05_06250 | ERK11276.1 | Phosphoglycerate kinase [Serratia fonticola AU-AP2C] | 98.191 | 387 | 0 | 100 |
| BBC05_13225 | KXJ03736.1 | glyceraldehyde-3-phosphate dehydrogenase [Serratia marcescens] | 97.321 | 336 | 0 | 100 |
| BBC05_19345 | ANM79803.1 | triose-phosphate isomerase [Serratia marcescens] | 99.216 | 255 | 0 | 100 |
| BBC05_13025 | SAY44498.1 | Fructose-bisphosphate aldolase class 1 [Serratia marcescens] | 99.427 | 349 | 0 | 100 |
| BBC05_06255 | EFE96163.1 | fructose-bisphosphate aldolase, class II [Serratia odorifera DSM 4582] | 96.936 | 359 | 0 | 100 |
| BBC05_04065 | SAY44197.1 | Transketolase [Serratia marcescens] | 97.527 | 283 | 0 | 100 |
| BBC05_06310 | KXJ00812.1 | ribose-phosphate isomerase A[Serratia marcescens] | 99.541 | 218 | 1.4E-154 | 100 |
| BBC05_10725 | ANM78414.1 | ribose -phosphate isomerase B [Serratia marcescens] | 97.744 | 133 | 1.24E-88 | 88 |
| BBC05_16715 | ANM76785.1 | ribulose-phosphate 3-epimerase [Serratia marcescens] | 99.111 | 225 | 6.77E-162 | 100 |
| BBC05_12275 | AFV36793.1 | carbonic anhydrase [Serratia sp. ISTD04] | 99.462 | 186 | 4.7E-132 | 100 |
| BBC05_02415 | AGE19010.1 | Citrate lyase [Serratia marcescens CUY86136.1] | 100 | 501 | 0 | 100 |
| BBC05_07410 | CUW02506.1 | Malate dehydrogenase [Serratia] | 97.756 | 312 | 0 | 100 |
| BBC05_15225 | CDS57382.1 | NAD-linked malate dehydrogenase [Serratia symbiotica] | 93.961 | 563 | 0 | 99 |
| BBC05_10660 | KGY55980.1 | malic enzyme [Serratia marcescens] | 99.868 | 759 | 0 | 100 |
| BBC05_13120 | CUW24027.1 | Fumarate hydratase class I [Serratia] | 96.533 | 548 | 0 | 100 |
| BBC05_12785 | SAY43017.1 | Fumarate hydratase class II [Serratia marcescens] | 99.57 | 465 | 0 | 100 |
| BBC05_05665 | CDG15027.1 | fumarate reductase [Serratia marcescens ] | 99.585 | 241 | 0 | 99 |
| BBC05_05670 | ANM78186.1 | fumarate reductase subunit C family protein [Serratia marcescens] | 99.225 | 129 | 1.59E-85 | 99 |
| BBC05_11935 | CDS57215.1 | succinyl-CoA synthetase alpha chain [Serratia symbiotica] | 97.931 | 290 | 0 | 100 |
| BBC05_11930 | CRH37540.1 | Succinyl-CoA ligase [ADP-forming] subunit beta [Pantoea ananatis] | 93.814 | 388 | 0 | 100 |
| BBC05_20805 | SAY42786.1 | Isocitrate dehydrogenase [NADP] [Serratia marcescens] | 99.754 | 406 | 0 | 97 |
| BBC05_11610 | SAY43401.1 | Aconitate hydratase [Serratia marcescens] | 98.876 | 890 | 0 | 100 |
| BBC05_15845 | CVF96668.1 | Pyruvate synthase [Serratia marcescens] | 99.065 | 1177 | 0 | 100 |
| BBC05_03135 | ANM80828.1 | phosphoenolpyruvate synthase regulatory protein [Serratia marcescens] | 98.901 | 273 | 0 | 100 |
| BBC05_03140 | ANM76094.1 | phosphoenolpyruvate synthase [Serratia marcescens] | 99.116 | 792 | 0 | 100 |
| BBC05_01275 | SAY45571.1 | Phosphoenolpyruvate carboxylase [Serratia marcescens] | 99.67 | 606 | 0 | 100 |
| BBC05_21020 | SAY42737.1 | Bicarbonate transporter BicA [Serratia marcescens] | 99.214 | 509 | 0 | 100 |
|  |  | **EPS biosynthesis Enzymes** |  |  |  |  |
| BBC05_01700 | SAY44294.1 | Glucokinase [Serratia marcescens] | 96.562 | 320 | 0 | 100 |
| BBC05_11790 | ETX39330.1 | phosphoglucomutase [Serratia marcescens BIDMC 44] | 99.817 | 547 | 0 | 100 |
| BBC05_09340 | SAY42029.1 | Galactose-1-phosphate uridylyltransferase [Serratia marcescens] | 96.286 | 350 | 0 | 100 |
| BBC05_15065 | KGY57505.1 | UDP-galactose-4-epimerase [Serratia marcescens] | 94.955 | 337 | 0 | 100 |
| BBC05_11335 | SAY43506.1 | UDP-glucose 6-dehydrogenase [Serratia marcescens] | 97.763 | 447 | 0 | 100 |
| BBC05_00160 | SAY41676.1 | Fructokinase [Serratia marcescens] | 96.382 | 304 | 0 | 100 |
| BBC05_12775 | SAY43015.1 | Mannose-6-phosphate isomerase [Serratia marcescens] | 98.724 | 392 | 0 | 100 |
| BBC05_15070 | KGY57506.1 | phosphomannomutase [Serratia marcescens] | 96.484 | 455 | 0 | 99 |
| BBC05_12775 | SAY43015.1 | Mannose-6-phosphate isomerase [Serratia marcescens] | 98.724 | 392 | 0 | 100 |
| BBC05_19510 | AAL78078.1 | glucosyl-transferase [Yersinia enterocolitica] | 99.611 | 257 | 0 | 100 |
| BBC05_13845 | SAY42616.1 | Glucans biosynthesis glucosyltransferase H [Serratia marcescens] | 99.531 | 852 | 0 | 100 |
| BBC05_19485 | KGY51962.1 | glycosyl transferase family 9 [Serratia marcescens] | 98.333 | 360 | 0 | 100 |
| BBC05_19765 | AGQ31694.1 | glycosyl transferase [Serratia liquefaciens ATCC 27592] | 77.419 | 279 | 3.54E-160 | 93 |
| BBC05_19775 | AHY07915.1 | polysaccharide biosynthesis protein [Serratia plymuthica] | 86.713 | 429 | 0 | 100 |
| BBC05_19785 | EZQ64369.1 | capsular polysaccharide translocation[Serratia marcescens BIDMC 81] | 100 | 369 | 0 | 98 |
| BBC05_23440 | ERH70632.1 | PTS sugar transporter subunit IIA [Serratia marcescens EGD-HP20] | 100 | 148 | 3.61E-104 | 100 |
| BBC05_04915 | KKO56354.1 | sugar (and other) transporter family protein [Serratia ureilytica] | 99.778 | 450 | 0 | 100 |
| BBC05_06740 | CUW23703.1 | PTS system mannose-specific EIIAB component [Serratia] | 86.111 | 144 | 8.36E-81 | 100 |
| BBC05_07750 | ANM80863.1 | glycosyl hydrolase 20, domain 2 family protein [Serratia marcescens] | 96.981 | 795 | 0 | 99 |
| BBC05_01605 | ANM80482.1 | glycogen/starch/alpha-glucan phosphorylases family protein [Serratia marcescens] | 99.376 | 801 | 0 | 100 |
| BBC05_11660 | CDG12485.1 | sugar ABC transporter, permease protein [Serratia marcescens subsp. marcescens Db11] | 99.153 | 354 | 0 | 100 |
| BBC05_12130 | SAY42246.1 | Biofilm dispersion protein BdlA [Serratia marcescens] | 98.848 | 434 | 0 | 100 |
| BBC05_13845 | SAY42616.1 | Glucans biosynthesis glucosyltransferase H [Serratia marcescens] | 99.531 | 852 | 0 | 100 |
| BBC05_13850 | ETX49065.1 | glucans biosynthesis protein G [Serratia marcescens BIDMC 50] | 99.8 | 500 | 0 | 100 |
| BBC05_13855 | KXJ03647.1 | glucans biosynthesis protein [Serratia marcescens] | 98.138 | 376 | 0 | 100 |
| BBC05_15035 | AID71053.1 | glycosyltransferase, family 2 protein [Aeromonas hydrophila] | 37.916 | 931 | 0 | 99 |
| BBC05_15050 | AID71050.1 | glycosyltransferase [Aeromonas hydrophila] | 53.203 | 359 | 3.74E-127 | 99 |
| BBC05_15060 | ANF30168.1 | dTDP-4-dehydrorhamnose 3,5-epimerase [Hafnia alvei] | 74.576 | 177 | 2.95E-100 | 100 |
| BBC05_15075 | KGY57507.1 | mannose-1-phosphate guanyltransferase [Serratia marcescens] | 95.992 | 474 | 0 | 100 |
| BBC05_15135 | CUW09536.1 | Glycosyl transferases group 1 [Serratia] | 52.895 | 380 | 1.65E-134 | 99 |
| BBC05_15145 | CUW09503.1 | UTP--glucose-1-phosphate uridylyltransferase [Serratia] | 90.236 | 297 | 0 | 100 |
| BBC05_15150 | AIA49534.1 | UTP-glucose-1-phosphate uridylyltransferase [Serratia sp. FS14] | 93.96 | 298 | 0 | 100 |
| BBC05_15890 | SAY43619.1 | PTS system mannose-specific EIIAB component [Serratia marcescens] | 98.746 | 319 | 0 | 100 |
| BBC05_15895 | AHM74410.1 | PTS system mannose-specific EIIC component [Yersinia enterocolitica LC20] | 92.105 | 266 | 2.53E-156 | 100 |
| BBC05_15900 | BAO34308.1 | mannose-specific enzyme IID component of PTS [Serratia marcescens SM39] | 100 | 280 | 0 | 100 |
| BBC05_16160 | AGO55685.1 | glycosyl transferase family 2 [Serratia plymuthica 4Rx13] | 83.333 | 300 | 0 | 100 |
| BBC05_16170 | AHY07910.1 | carbohydrate-binding protein CenC [Serratia plymuthica] | 73.844 | 627 | 0 | 100 |
| BBC05_16330 | KXJ03416.1 | diguanylate cyclase [Serratia marcescens] | 92.5 | 400 | 0 | 100 |
| BBC05_19035 | AHM73177.1 | D-galactose/ D-glucose-binding protein [Yersinia enterocolitica LC20] | 93.939 | 330 | 0 | 100 |
| BBC05_06060 | SAY44415.1 | GDP-mannose pyrophosphatase NudK [Serratia marcescens] | 98.421 | 190 | 3.35E-137 | 99 |
